# Mobile cognitive assessment demonstrates diagnostic equivalence to MMSE and MoCA scales in Alzheimer’s disease screening

**DOI:** 10.3389/fneur.2026.1759621

**Published:** 2026-02-25

**Authors:** Yuezhou Zhang, Qing Chen, Hao Xie, Wen Chang, Shiqin Huang, Min Zhang

**Affiliations:** 1 Department of Respiratory and Critical Care Medicine, The First Affiliated Hospital of Chongqing Medical University, Chongqing, China; 2 Department of Outpatient, Sichuan Provincial People’s Hospital, School of Medicine, University of Electronic Science and Technology of China, Chengdu, China; 3 Department of Neurology, Xindu District Traditional Chinese Medicine Hospital, Chengdu, Sichuan, China

**Keywords:** Alzheimer’s disease, cognitive screening, machine learning, MCAS, mild cognitive impairment, mobile cognitive assessment

## Abstract

**Introduction:**

Alzheimer’s disease (AD), the most common neurodegenerative disorder, poses significant challenges for early screening due to the clinical and environmental constraints of traditional neuropsychological assessments.

**Methods:**

This study developed a mobile terminal-based cognitive assessment system (mCAS) and prospectively validated its screening efficacy through a diagnostic trial. We recruited 63 memory clinic patients (aged 20–75 years), all of whom independently completed mCAS testing after undergoing standardized MMSE and MoCA evaluations. Through a systematic review of 10 existing mild cognitive impairment (MCI) screening tools, we extracted 25 test items to construct the assessment framework.

**Results:**

Our results demonstrated that, under the optimal Gradient Boosting model, mCAS achieved an area under the curve (AUC) of 0.884 for discriminating MCI while maintaining diagnostic equivalence in sensitivity compared to conventional instruments (*p* > 0.05 in all pairwise comparisons). Specificity was significantly lower than MoCA only for MCI identification (*p* = 0.027).

**Discussion:**

The system’s core innovations include: (1) A multimodal digital assessment framework that overcomes the environmental limitations of conventional scales; (2) Self-administration capability in non-medical settings; and (3) A dynamic cognitive baseline model to facilitate longitudinal monitoring. mCAS provides a convenient screening solution for early AD detection, with significant potential particularly in resource-limited regions. Future multicenter validation and biomarker integration studies are warranted.

## 1 Introduction

Alzheimer’s disease (AD) represents a major global public health challenge, with its disability rate and disease burden escalating annually (1). According to World Health Organization statistics, a new case of dementia occurs every 3 s worldwide, of which AD accounts for 60–70% (2). It is projected that the global number of AD patients will exceed 150 million by 2050 (3). Epidemiological studies reveal that patients with AD exhibit pathological changes lasting 10–20 years before clinical symptoms manifest (4), including β-amyloid (Aβ) deposition (5) and abnormal tau protein phosphorylation (6). This preclinical phase is recognized as the “golden window for intervention” in disease management.

However, traditional screening tools such as the Mini-Mental State Examination (MMSE) and Montreal Cognitive Assessment (MoCA) (7, 8), despite their widespread use, face inherent limitations that severely constrain early screening efficiency (9). First, these scales require administration by specialized clinicians, are time-consuming (10), and are susceptible to environmental interference, resulting in community screening coverage below 35% and a missed diagnosis rate as high as 42% (11). Second, their static assessment paradigm can only capture cognitive status at a single timepoint, failing to dynamically track trajectories of subtle cognitive decline—a critical factor for monitoring disease progression and evaluating intervention efficacy (12).

In recent years, mobile health (mHealth) technology has created new opportunities for revolutionizing AD screening (13). Although some studies have attempted to digitize paper-based scales, most tools remain confined to simple content migration without fully leveraging the cognitive assessment potential of mobile-specific behavioral data (e.g., touchscreen trajectories, vocal micro-features, and accelerometer signals) (14). Recent advances in cognitive neuroscience demonstrate that behavioral metrics—including fine motor control (e.g., fluency and coordination in touchscreen operations), reaction time variability (e.g., latency differences during task-switching), and subtle vocal spectrum changes (e.g., tonal stability and semantic coherence)—show significant correlations with core AD biomarkers such as hippocampal atrophy and Aβ deposition (15–17). Multimodal integration of behavioral data with biomarkers can substantially enhance early screening efficacy (18–20).

However, no existing mobile tool comprehensively captures these multimodal behavioral indicators in a clinically validated framework. Against this backdrop, we aimed to develop a mobile terminal-based cognitive assessment system (mCAS) to overcome the static limitations of traditional scales. Through systematic review of existing AD screening tools, we integrated 25 multimodal test items covering eight cognitive domains (executive function, attention, visuospatial ability, etc.) and constructed a dynamic cognitive baseline model using machine learning algorithms. The system’s innovations include (1): enabling self-assessment in non-medical settings whereby patients can independently complete 5-min rapid tests, reducing reliance on specialists; and (2) supporting longitudinal dynamic monitoring through backend storage of results, providing a data foundation for personalized cognitive trajectory analysis. Validated by a prospective diagnostic trial, this research delivers a scalable digital solution for early AD screening, particularly applicable in resource-limited regions, while establishing a technical framework for future multimodal studies integrating biomarkers and behavioral data.

## 2 Methods

### 2.1 Participants

All participants were recruited from the memory clinic at Sichuan Provincial People’s Hospital. Inclusion criteria were as follows (1): fluent in Chinese and able to accurately read and write Chinese (2); no comorbid diseases affecting cognitive function and no history of psychiatric illness; and (3) willingness to participate in this study. All participants underwent MMSE and MoCA assessments, followed by mCAS testing. All participants provided written informed consent. This study was approved by the Institutional Review Board of Sichuan Provincial People’s Hospital.

### 2.2 Mobile-based cognitive assessment system

**Figure 1:**
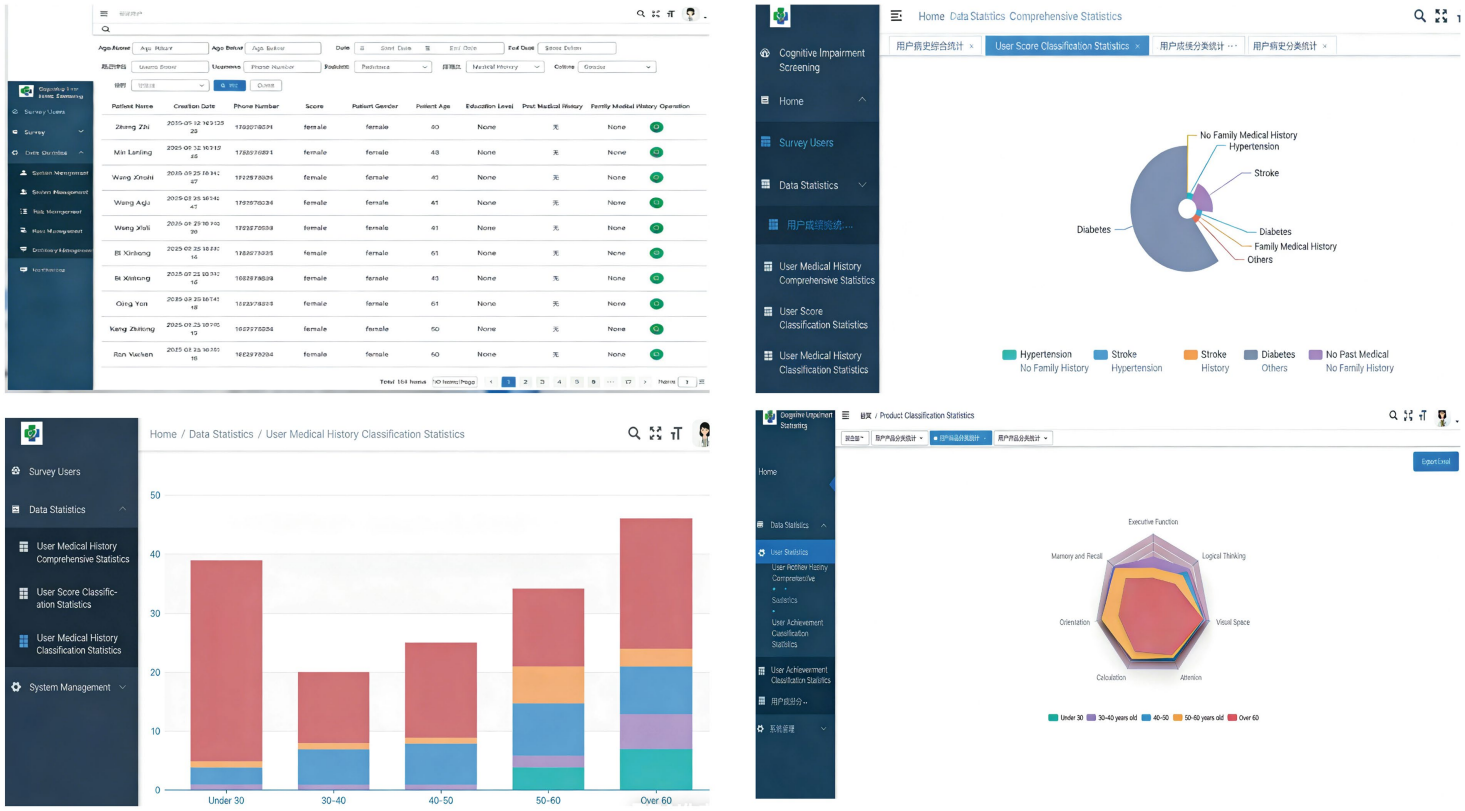
Display of the mCAS operating interface.

**Figure 2:**
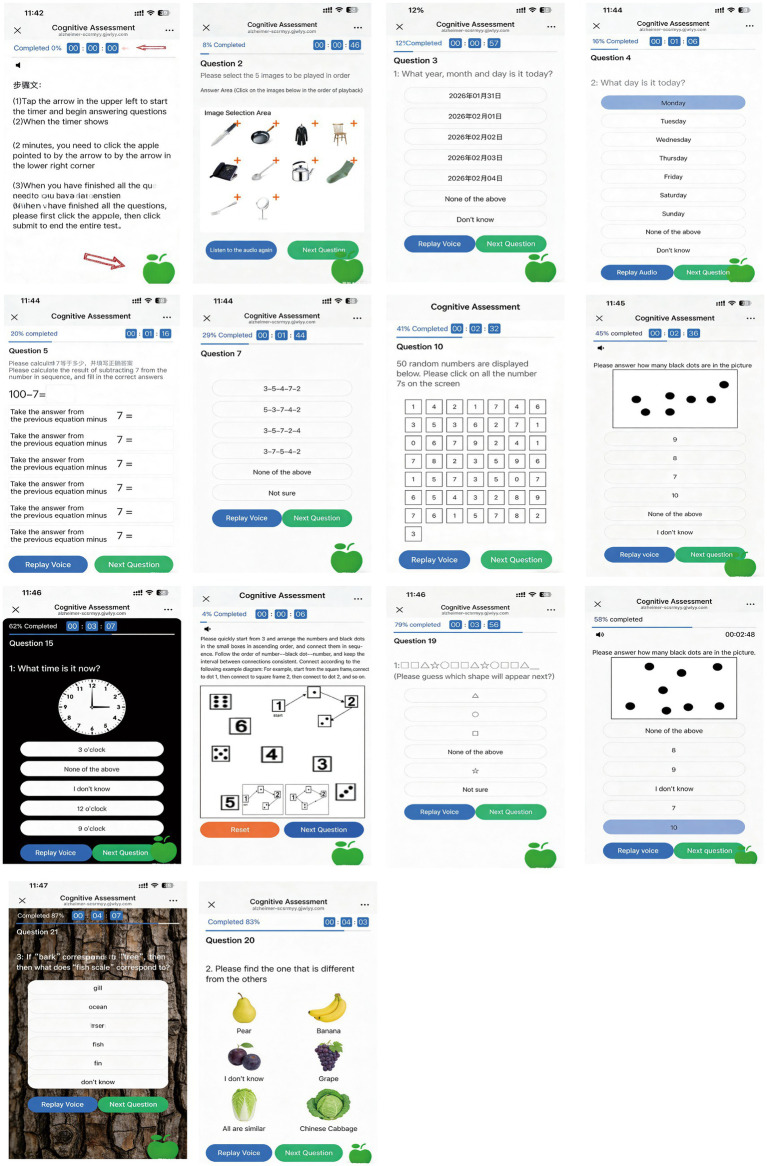
Display of the mCAS patient-side interface.

The mobile terminal utilized HTML5 + CSS3 for interface presentation; the management backend web portal employed the VUE + Element framework for page display with ECharts for visual data analytics; and backend services adopted Spring Boot + MyBatis for data processing and MySQL for database storage. To develop mCAS, the research team first conducted a systematic review of 10 existing MCI screening tools. After eliminating redundant items, 25 test components were extracted and categorized into eight cognitive domains: executive function, immediate and delayed recall, orientation, calculation, attention, visuospatial ability, logical thinking, and language ability. Using the Delphi method, test items and scoring criteria (including point allocation and evaluation standards) were established for each domain. The scale has a maximum score of 35 points, where higher scores (0–35 range) indicate better cognitive function. The entire assessment can be completed within 5 min (Figures 1, 2).

### 2.3 Montreal Cognitive Assessment

The Montreal Cognitive Assessment (MoCA) was developed to provide a rapid, sensitive, and user-friendly screening instrument for cognitive impairment, with particular emphasis on detecting MCI. The finalized revised version encompasses eight core cognitive domains. Empirical evidence confirms its excellent test–retest reliability (ICC >0.85) and robust predictive validity for both MCI and AD, with positive predictive values (PPV) exceeding 82% and negative predictive values (NPV) surpassing 89% in validation cohorts (8).

### 2.4 Mini-Mental State Examination

The Mini-Mental State Examination (MMSE) is a widely used cognitive screening instrument in both clinical and research settings. Originally developed by Folstein et al. in 1975, this gold standard assessment primarily evaluates cognitive impairment in older adults, including AD and other forms of dementia. The MMSE comprises 30 standardized items organized across five core cognitive domains: orientation, registration, attention/calculation, recall, and language (21).

### 2.5 Statistical analysis

Raw data underwent preprocessing by removing cases with missing values. The dataset was then partitioned into training and validation sets at a 7:3 ratio for model construction and optimization. Five machine learning algorithms were implemented: Random Forests (RF), Gradient Boosting (GB), Support Vector Machine (SVM), k-Nearest Neighbors (k-NN), and Neural Network (NN), with automated hyperparameter tuning via Python scripts to enhance model performance. Model predictive efficacy was evaluated by comparing performance metrics including accuracy, area under the curve (AUC), precision, *F*_1_-score, and confusion matrix outcomes to identify the optimal model. All machine learning analyses were performed in Python 3.10.4, while statistical comparisons employed one-way ANOVA or *t*-tests for continuous variables and *χ*^2^ tests for categorical variables.

## 3 Results

### 3.1 Baseline characteristics

This study prospectively enrolled 63 memory clinic patients aged 20–75 years. Thirty-four participants (55.3%) were in the <60 years cohort, demonstrating significantly different biomarker profiles compared to 29 patients (44.7%) in the ≥60 years group. Gender distribution showed male predominance (41 males, 68.3% vs. 22 females, 31.7%). Educational stratification revealed 29 participants (46.0%) with tertiary education (ISCED levels 5–8), 14 (22.2%) with secondary education (ISCED 3–4), and 20 (31.7%) with primary education or below (ISCED 0–2). Educational attainment significantly correlated with baseline MoCA scores and showed differential distribution across age cohorts (≥60 years: 65.5% primary education vs. <60 years: 23.5%) (Table 1).

**Table 1:** Baseline characteristics.

Characteristic	Number (%)
Age	
<60	34 (55.3%)
≥60	29 (44.7%)
Gender	
Male	41 (68.3%)
Female	22 (31.7%)
Education level	
Junior school education or lower	29 (46%)
High school education	14 (22.2%)
College education or higher	20 (31.7%)

### 3.2 Performance comparison of machine learning models

**Figure 3:**
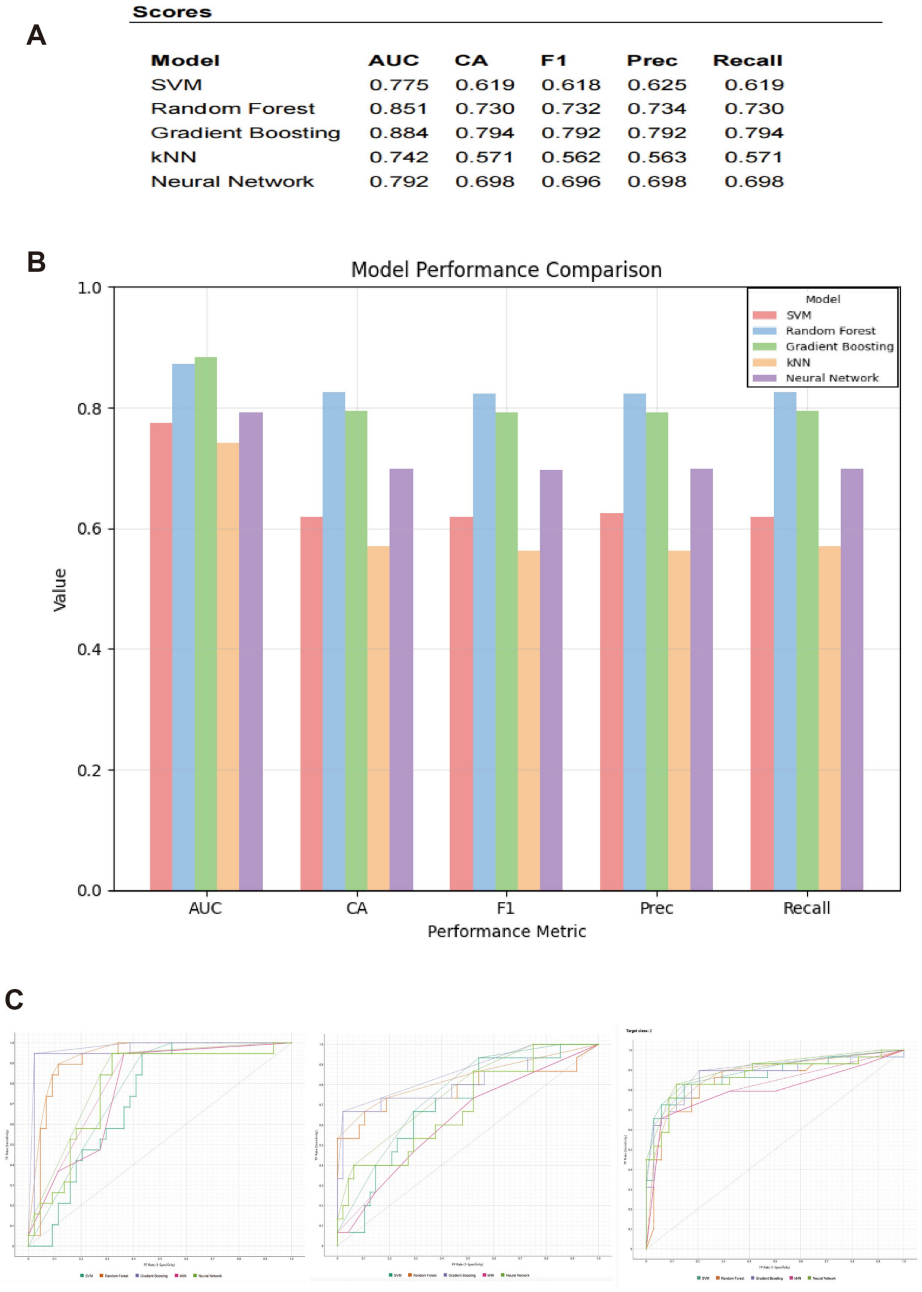
Performance comparison of machine learning models and ROC analysis for AD diagnosis. **(A)** Performance metrics for five machine learning classifiers—SVM, RF, GB, k-NN, and NN—including AUC, CA, *F*_1_-score, precision, and recall. **(B)** Bar plot illustrating the comparative performance of the five models across all evaluation metrics. **(C)** ROC curves of the five machine learning models based on the three cognitive assessment scales used in this study for AD identification.

Figure 3 demonstrates the discriminatory efficacy of five machine learning models—Support Vector Machine (SVM), Random Forest (RF), Gradient Boosting (GB), Neural Network (NN), and k-Nearest Neighbors (k-NN)—through validation set performance comparison. Analysis of AUC and classification accuracy (CA) revealed AUC values ranging from 0.742 to 0.884 and CA values ranging from 0.571 to 0.794 across all models. The GB model exhibited optimal overall performance (AUC: 0.884 ± 0.021; CA: 0.794 ± 0.018), followed by RF (AUC: 0.851 ± 0.024; CA: 0.730 ± 0.022), NN (AUC: 0.792 ± 0.026; CA: 0.698 ± 0.025), SVM (AUC: 0.775 ± 0.028; CA: 0.619 ± 0.030), and k-NN (AUC: 0.742 ± 0.031; CA: 0.571 ± 0.032). Statistical validation via DeLong’s test (AUC comparison) and McNemar’s test (CA comparison) confirmed GB’s significant superiority over RF (AUC Δ = 0.033, *p* = 0.008; CA Δ = 0.064, *p* = 0.003), with all inter-model differences reaching *p* < 0.01. Therefore, GB was selected as the final algorithm for subsequent analyses.

### 3.3 Sensitivity and specificity

**Figure 4:**
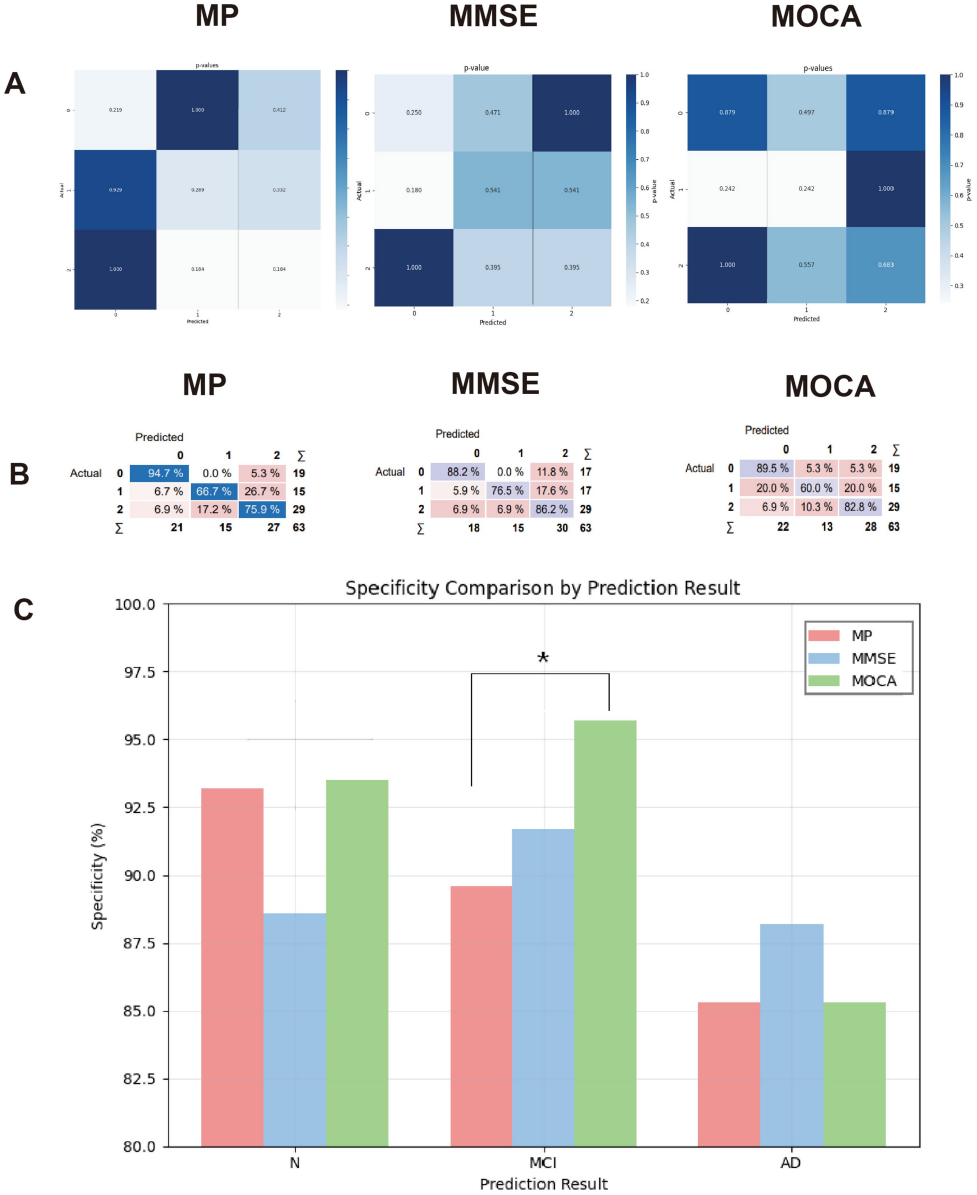
Comparative performance of mCAS, MMSE, and MoCA in AD identification. **(A)** Pairwise confusion matrices for mCAS vs. MMSE, mCAS vs. MoCA, and MMSE vs. MoCA. Numbers indicate *p*-values; the *x*-axis represents predicted values and the *y*-axis represents actual values (0 = CN, 1 = MCI, 2 = AD). Diagonal cells denote sensitivity comparison between scales for each category; *p* < 0.05 indicates statistical significance. **(B)** Confusion matrices of each scale for categories 0, 1, and 2; diagonal cells indicate category-specific sensitivity. **(C)** Specificity comparison of the three scales, with a significant difference between mCAS and MoCA in identifying MCI (^*^p < 0.05).

Based on validation results from the optimal GB model, mCAS exhibited heterogeneous diagnostic performance compared to the standardized instruments—MMSE and MoCA—across three diagnostic categories: cognitively normal (CN), MCI, and AD. Sensitivity analysis revealed no statistically significant differences between mCAS and MoCA for CN (McNemar’s test: *p* = 0.219), MCI (*p* = 0.289), or AD (*p* = 0.184), nor between mCAS and MMSE for corresponding groups (CN: *p* = 0.250; MCI: *p* = 0.541; AD: *p* = 0.395). However, specificity comparisons revealed a critical divergence: for MCI identification, mCAS specificity was significantly lower than MoCA [89.6% (95% CI, 86.2–92.3%) vs. 95.7% (95% CI, 93.1–97.5%), *χ*^2^ = 4.92, *p* = 0.027], whereas no significant differences were observed between mCAS and MMSE [91.4% (88.7–93.6%), *p* = 0.360] or between MoCA and MMSE (*p* = 0.144), confirming a specificity limitation of mCAS in MCI screening (Figure 4).

## 4 Discussion

The escalating global burden of AD necessitates innovative solutions to overcome the limitations of traditional cognitive screening tools (22). Although the MMSE and MoCA remain clinical standards, their reliance on professional administration severely limits accessibility, particularly in rural and resource-limited settings (23). The mCAS developed in this study addresses this gap by enabling self-administered testing and remote clinical monitoring, thereby advancing equitable early AD screening. Our findings highlight both the potential and challenges of digital cognitive assessment in real-world implementation.

The mCAS demonstrated 66.7% sensitivity for MCI, showing no statistical difference from MMSE (60.0%) or MoCA (76.5%) (*p* > 0.05). For AD detection, mCAS achieved 75.2% sensitivity, comparable to MMSE (82.8%) and MoCA (86.2%), supporting its potential as a community-level frontline screener. Specificity analysis revealed a statistically significant difference only between mCAS and MoCA in MCI identification [89.6% (95% CI, 86.2–92.3%) vs. 95.7% (93.1–97.5%), *χ*^2^ = 4.92, *p* = 0.027], while mCAS-MMSE [91.4% (88.7–93.6%)] and MoCA-MMSE comparisons showed equivalence (*p* > 0.05). This specificity gap may elevate false-positive referrals in MCI screening, potentially causing unnecessary anxiety or overtreatment. The discrepancy likely stems from mCAS’s reliance on static behavioral metrics (e.g., reaction time, task accuracy) rather than dynamic context-sensitive features (e.g., micro-fluctuations in motor coordination during multitasking) (24). Unlike traditional scales containing neuropathologically validated domain-specific tasks, current mCAS design lacks granularity to distinguish AD-specific decline from age-related or stress-induced impairment (25).

A critical finding is that neither MMSE, MoCA, nor mCAS demonstrate optimal sensitivity for MCI, which is a significant limitation, as MCI represents the critical therapeutic window for disease-modifying interventions. This may reflect insufficient test complexity: tasks such as delayed recall or visuospatial puzzles fail to adequately challenge executive functions or working memory to detect subtle deficits. Future versions could enhance MCI detection by incorporating adaptive difficulty algorithms or performance-based gamified paradigms.

Although the GB model outperformed alternatives, its failure to achieve AUC >0.9 highlights feature engineering constraints. Behavioral data (e.g., touch trajectories, reaction times) were analyzed as static aggregates (mean latency, path length), overlooking temporal dynamics (e.g., acceleration patterns during task-switching, micro-pauses reflecting cognitive effort). For instance, early AD patients may maintain normal aggregate accuracy while exhibiting abnormal reaction time variability under high cognitive load—patterns missed by current models (26).

Emerging deep learning architectures could address these gaps: long short-term memory (LSTM) networks can better capture sequential dependencies in touch interactions (27), while Transformer models analyze multimodal data streams (e.g., synchronization of vocal pitch changes and screen-tapping rhythms) to identify neurodegenerative biomarkers. Federated learning frameworks could continuously optimize models across populations without compromising data privacy—an approach crucial for global deployment.

### 4.1 Population bias and generalizability challenges

Our study population characteristics introduced significant bias: younger age (55.3% <60 years) (25) and higher education (46% tertiary) may overestimate specificity due to lower AD prevalence and greater cognitive reserve (28). Elevated education levels might mask early decline through the “cognitive reserve effect”.

Enhancing generalizability requires prioritized inclusion of older (≥65 years) and lower-education cohorts. Adaptive interface designs (e.g., dialect-based voice guidance, icon-based task prompts) could reduce literacy-related barriers. Longitudinal tracking of mCAS performance against gold-standard biomarkers (amyloid-PET, CSF tau) in multisite cohorts will clarify its prognostic value across socioeconomic and cultural contexts (29).

### 4.2 Public health integration and scalability

The mCAS shows unique potential for large-scale screening. Its continuous real-world data capture enables personalized cognitive baselines—revolutionizing AD monitoring beyond static scales. For example, longitudinal decline in daily problem-solving abilities may predict MCI-to-AD conversion earlier than annual clinical assessments. Integration with telemedicine platforms could automate risk alerts, triggering timely follow-up for high-risk individuals.

However, clinician-dependent interpretation remains a bottleneck. Embedded explainable AI (XAI) modules generating intuitive risk reports (e.g., “75% AD risk: 6-month decline in spatial navigation accuracy”) could empower primary care physicians to act without specialist input. Cost-effectiveness analyses are essential: while mCAS reduces infrastructure costs, its long-term value depends on balancing false-positive referrals against delayed diagnosis in resource-limited settings.

### 4.3 Future directions

Several directions warrant future investigation. First, multimodal biomarker integration—combining mCAS with wearables (e.g., smartwatches detecting sleep fragmentation) or minimally invasive biomarkers (e.g., plasma p-tau217)—could develop composite risk scores with enhanced diagnostic precision. Second, cross-ethnic validation is needed to test mCAS in populations with AD genetic risk variants (e.g., APOE ε4 carriers in African/Asian cohorts) to evaluate cross-cultural robustness. Third, longitudinal data can be collected from each patient to develop individualized cognitive trajectory models, which can be analyzed using linear mixed models or growth curve models. Lastly, regulatory and ethical frameworks must be established for digital cognitive tools to ensure data security, algorithmic transparency, and equitable access.

## 5 Conclusion

In conclusion, mCAS represents a transformative step toward accessible AD screening; however, its clinical translation requires addressing technical, demographic, and implementation challenges. By integrating AI advances, prioritizing inclusive design, and fostering cross-disciplinary collaboration, mobile cognitive assessment can shift AD care from reactive diagnosis to proactive, personalized prevention.

## Data Availability

The raw data supporting the conclusions of this article will be made available by the authors, without undue reservation.
